# ITO-Induced Nonlinear Optical Response Enhancement of Titanium Nitride Thin Films

**DOI:** 10.3390/nano14121040

**Published:** 2024-06-17

**Authors:** Peng Lu, Tingzhen Yan, Jialei Huang, Tian Xing, Hao Liu, Zhaoxia Han, Xueke Xu, Chunxian Tao

**Affiliations:** 1 Engineering Research Center of Optical Instrument and System, Ministry of Education and Shanghai Key Lab of Modern Optical System, University of Shanghai for Science and Technology, Shanghai 200093, China; 2135061716@st.usst.edu.cn (P.L.);; 2 Department of Printing and Pack Aging Engineering, Shanghai Publishing and Printing College, No. 100 Shuifeng Road, Shanghai 200093, China; tzyancn@163.com; 3 Heng Mai Optics and Fine Mechanics (Hang Zhou) Co., Ltd., Hangzhou 311421, China

**Keywords:** TiN, ITO, Z-scan, nonlinear optics, magnetron sputtering

## Abstract

A series of TiN/ITO composite films with various thickness of ITO buffer layer were fabricated in this study. The enhancement of optical properties was realized in the composite thin films. The absorption spectra showed that absorption intensity in the near-infrared region was obviously enhanced with the increase of ITO thickness due to the coupling of surface plasma between TiN and ITO. The epsilon-near-zero wavelength of this composite can be tuned from 935 nm to 1895 nm by varying the thickness of ITO thin films. The nonlinear optical property investigated by Z-scan technique showed that the nonlinear absorption coefficient (β = 3.03 × 10^−4^ cm/W) for the composite was about 14.02 times greater than that of single-layer TiN films. The theoretical calculations performed by finite difference time domain were in good agreement with those of the experiments.

## 1. Introduction

Nonlinear optics mainly studies the nonlinear phenomena, theories, and applications of media under strong light, which originated from the discovery of the Bubbles effect and the Kerr effect [1]. The emergence of lasers has provided the necessary high-intensity and strong coherence beams for nonlinear optical research, enabling its rapid development and playing a very important role in modern optoelectronic functional devices and systems. As a result, it has been widely applied in fields such as optical communication, all optical information processing and storage, spectroscopy technology, quantum information technology, etc. [2,3,4,5,6]. The extensive application prospects of nonlinear optical technology highlight the importance of nonlinear optical materials [7].

Traditional noble metals exhibit excellent nonlinear optical properties due to their high carrier concentration and surface electron cloud density, resulting in enhanced resonance absorption nonlinearity [8,9]. Due to the significant loss issue of metal materials, especially in the visible and ultraviolet regions, almost all highly conductive metals have significant losses in the optical frequency band. Two-dimensional materials represented by graphene exhibit unique optoelectronic properties due to their unique structure and quantum confinement effects. Their nonlinear optical properties and applications are also extensively studied by researchers [10,11,12]. The low thermal, chemical, and mechanical stability of saturable absorption based on noble metals and new two-dimensional materials limits its application in extreme working environments [13].

To overcome the stability issues of noble metals and two-dimensional materials, nitride-based and transparent conductive oxides (TCO) materials have been explored in recent years as alternative materials for the engineering of plasmonic resonances and for the design of metamaterials in the near-infrared spectral range [14]. As an excellent refractory and wear-resistant material, TiN film is gradually being discovered and applied in optoelectronic devices by researchers due to its excellent stability and suitable localized surface plasmon resonance response bands [15].

TCO has been widely used in fields such as solar cells, flat panel displays, organic light-emitting diodes, low radiation glass, transparent thin-film transistors, and flexible electronic devices due to its excellent conductivity and high transmittance in the visible and near-infrared regions [16]. Recent studies have found that in addition to excellent conductivity, TCO also possesses some remarkable optical properties, such as nonlinear optical effects [17]. From the near-infrared to mid infrared region, the real part of the dielectric constant of the TCO film can be close to zero. According to the electromagnetic field boundary conditions, TCO material can locally enhance the electric field in the ENZ region, thereby achieving efficient electro-optic modulation [18]. Recent studies have shown that the deposition of gold nanoparticles with different particle densities on ITO substrates can generate second harmonic waves induced by coherent light [19]. ITO/Sn composite films with thickness dependence have also been shown to have strong nonlinear optical response [20]. In addition, simple pretreatment of ITO-Au nanocomposite arrays by self-assembled nanolithography (NSL), as well as pretreatment under annealing conditions, also showed significant two-photon absorption (TPA) saturation effects [21]. ITO-based composite films show amazing potential in nonlinear optics. In addition, materials with high nonlinear saturable absorption coefficients can be used as saturable absorption materials. Saturable absorption materials [22], which can convert continuous laser waves into laser pulses, are expected to be ideal candidates for all-optical modulation devices at optical communication wavelengths.

In this study, we investigated the nonlinear optical response of TiN/ITO composite materials using the typical Z-scan experiments. Our experiments provide a strategy to tune the ENZ region in TiN based thin films. We have also investigated the effects of ITO buffer layer on the structure and optical properties of TiN thin films. In addition, the evolution of internal defects and the change of optical properties in TiN/ITO composites films are verified by the FDTD method.

## 2. Materials and Methods

Before deposition, the K9 glass substrates were cleaned in acetone, ethanol, and deionized water with ultrasonic waves for 30 min and then dried with a nitrogen gas stream. The chamber was pumped to a base pressure of 1 × 10^−4^ Pa before deposition with the baking temperature of 350 °C. ITO films were grown by magnetron sputtering from sintered ITO ceramics target (99.99%) containing 10 wt % SnO_2_. High-purity argon and oxygen were used as the sputtering and reactive gases, respectively. Film growth was carried out in a mixture of argon (40%) and oxygen (60%) and at a constant working pressure of 0.15 Pa. The ITO layer was deposited with the thickness of 50 nm, 100 nm, 200 nm, and 300 nm, respectively. The thickness of the film was monitored by an in situ quartz crystal microbalance. To guarantee the uniformity of the film thickness, all substrates were set on the fixture with the same radius of the circle.

The TiN thin film was deposited on the ITO buffer layer with the thickness of 50 nm by magnetron reactive sputtering using a Ti target (99.99%). Film growth was carried out in a mixture of argon (30%) and nitrogen (70%) and at a constant working pressure of 0.5 Pa with a baking temperature of 300 °C and bias voltage of 150 V, respectively. From bottom to top in TiN/ITO composite, the bottom layer is a K9 glass substrate, the middle layer is ITO films with different thicknesses, and the top layer is TiN with a thickness of 50 nm. For comparison, TiN thin film and ITO thin film deposited on bare substrate with the same thickness were also used in this experiment.

The structures and crystallinity of the samples were characterized X-ray diffraction (XRD) on a Bruker (Billerica, MA, USA) AXS/D8 advanced system with a scanning range of 10–80° and a step size of 5°/min, a tube voltage of 40 KV, and a tube current of 15 mA. An atomic force microscope (AFM, XE-100, Park System, Suwon, Republic of Korea) was used to characterize the root mean square (RMS) roughness of the samples, and the surface morphology of the samples was analyzed using scanning electron microscopy (SEM) (a TESCAN MIRA LMS Instrument from the Czech Republic, 15 KV accelerating voltage, 7 nm working distance). A UV-Vis-NIR dual-beam spectrophotometer was used to measure the absorption of the samples (Lambda1050, PerkinElmer, Waltham, MA, USA). In order to investigate the nonlinear optical properties and obtain the saturable absorption (SA) coefficients of the samples, we constructed an optical aberration (self-focusing) measurement device based on the single-beam open-aperture (OA) Z-scan technique. The key parameters of the system are as follows: the measurement light source is a mode-locked picosecond laser (Menlo Systems Martinsried/Munich, Germany, the repetition rate of 20 MHz, pulse width 2 ps) with a wavelength of 1550 nm, and its Gaussian beam was focused by a lens with a focal length of 15 nm. All the measurements were carried out at room temperature.

## 3. Results and Discussion

**Figure 1:**
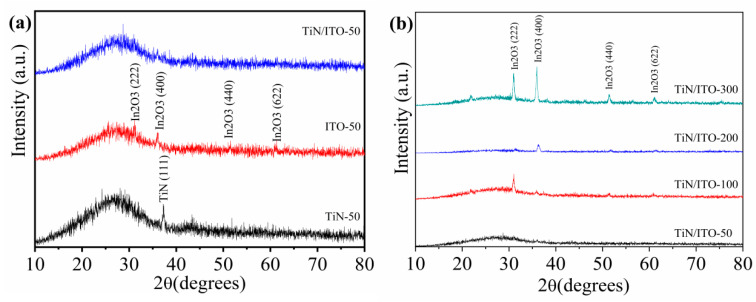
(**a**) XRD patterns of the single-layer TiN, single-layer ITO, and 50 nm TiN/ITO composite film. (**b**) XRD patterns of the TiN/ITO composite films with different ITO thicknesses.

Figure 1 shows the XRD patterns of single-layer TiN, single-layer ITO, and TiN/ITO composite films with different ITO thicknesses. In our experiments, we set the scanning range from 10° to 80° with a step size of 5°/min. According to the figure, it can be seen that the single-layer TiN film has a diffraction peak at about 37 ° (2θ), corresponding to the TiN (111) crystal plane, indicating that the TiN film exhibits a (111) plane preferred orientation (PDF#38-1420). While the single-layer ITO sample exhibits diffraction peaks at 31°, 36°, 51 °, and 61°(2θ), corresponding to In_2_O_3_ (222), (400), (440), and (622) crystal planes, respectively (PDF#06-0416). With the thickness of ITO increasing, the diffraction peak intensity increases, and an additional obvious diffraction peak also appears at 21°(2θ), corresponding to the In_2_O_3_ (211) crystal plane. For TiN/ITO composite film samples, when the ITO film thickness is thin, the composite film sample almost exhibits an amorphous state. With the increase of ITO film thickness, the diffraction peak intensity of TiN/ITO composite film shows an increasing trend on different diffraction surfaces. The preferred orientation of composite films also varies with the thickness of the ITO film layer. As the ITO thickness is 100 nm, TiN/ITO composite film exhibits (222) plane preferred orientation, and as the thickness of ITO film increases to 200 nm, TiN/ITO composite film exhibits (400) plane preferred orientation. When the ITO film thickness increases to 300 nm, all diffraction peak diffraction intensities in TiN/ITO composite film are enhanced.

**Figure 2:**
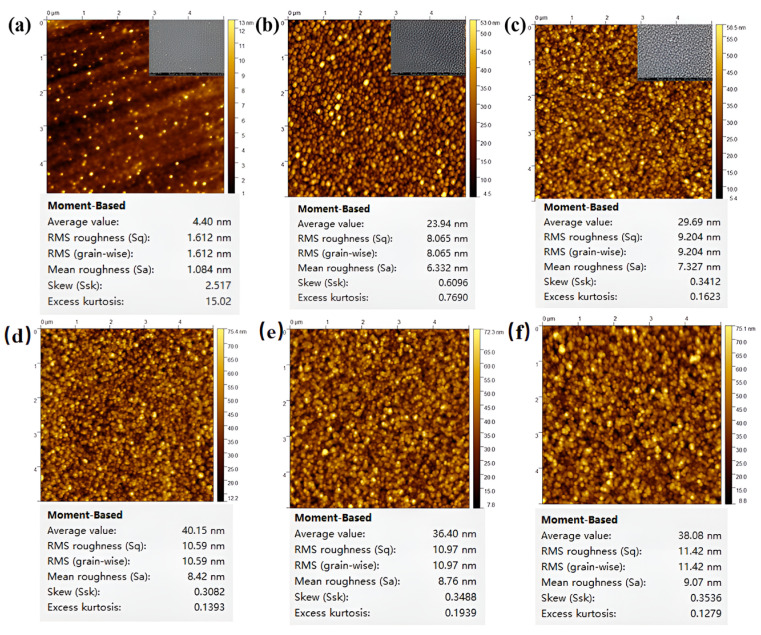
SEM and AFM micrographs of (**a**) single-layer 50 nm TiN, (**b**) single-layer 50 nm ITO, (**c**) TiN/ITO 50 nm composite films, (**d**) TiN/ITO 100 nm composite films, (**e**) TiN/ITO 200 nm composite films, (**f**) TiN/ITO 300 nm composite films.

The surface roughness was investigated in the tapping mode by atomic force microscopy (AFM) (XE-100, Park Systems) with scanning area for 5 μm × 5 μm. The force constant and frequency of probe are 42 N/m, and 330 kHz, respectively. The typical tip radium is about 2 nm. Figure 2 shows AFM micrographs of the prepared specimen. The samples are single-layer TiN, single-layer 50 nm ITO, and TiN/ITO composite films with different ITO thickness films, corresponding to root mean square roughness of 1.612 nm, 8.065 nm, and 9.204 nm, 10.590 nm, 10.970 nm, and 11.42 nm, respectively. According to Figure 2, the surface of single-layer TiN film is very smooth with a low surface roughness value, while the surface of ITO film is much rougher than that of TiN film, with a root mean square roughness nearly four times that of TiN film. For TiN/ITO composite films, the surface roughness is higher than that of either single TiN layer or ITO single-layer for the presence of interfaces between the films leads to an increase in surface roughness. The surface roughness of the composite film increases with the thickness of the ITO film layer increasing. The SEM images further verified the variation trend of surface roughness of the sample.

**Figure 3:**
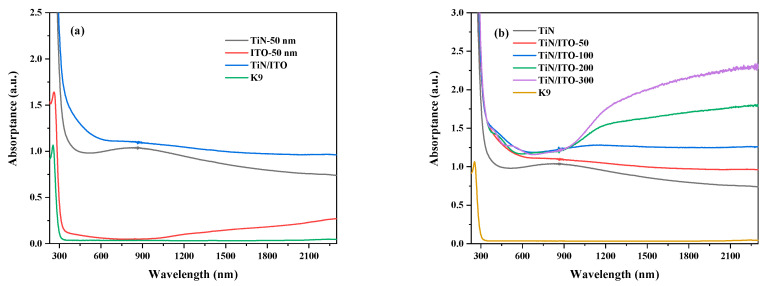
Absorption curves of (**a**) single-layer TiN, single-layer ITO, and TiN/ITO composite films. (**b**) single-layer TiN and TiN/ITO composite films with different ITO thicknesses.

Figure 3 shows the absorption curves of single-layer TiN, single-layer ITO, and TiN/ITO composite films with different ITO thicknesses. According to Figure 3a, the single-layer TiN film has a significantly widened absorption band in the regions from visible to near-infrared, corresponding to its plasma absorption peak [23]. However, the absorption intensity of the single-layer ITO film exhibits very low values in the visible light region, and no obvious absorption peak was observed, while in the near-infrared region, the absorption intensity increases overall, and the absorption intensity increases with the increase of wavelength. The main reason is that the plasma absorption edge of ITO leads to an increase in absorption intensity. For TiN/ITO composite films, the total absorption intensity increases, and there is no obvious localized plasma absorption peak. With the thickness of the ITO film layer increasing, the absorption in the near-infrared region is significantly enhanced, as shown in Figure 3b.

**Figure 4:**
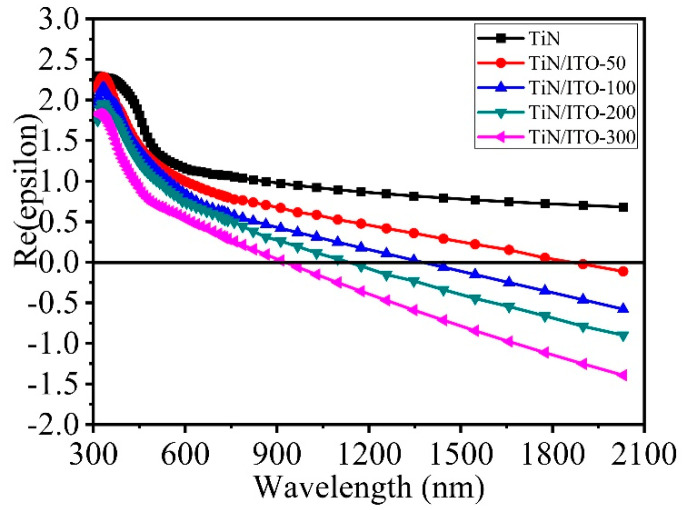
The permittivity of single-layer TiN film and TiN/ITO composite films with real parts. The black line is the horizontal tangent with Re(epsilon) 0.

The dielectric constant characteristics of the samples were characterized through an elliptical polarization spectrometer in the experiment. Figure 4 shows the dielectric constant spectrum of the sample after fitting the ellipsometer test data based on the Drude and Drude Lorentz models [24,25]. The interaction with the electromagnetic field is described using the Drude model, and the absorption of photons is described using the Lorentz harmonic oscillator model. The root mean square error of fitting the data measured by the ellipsometer is less than 5, and the fitting data fits the actual data peak shape. The dielectric constant of TiN/ITO based thin films can be calculated using the following formula:

$$\varepsilon \left(\omega \right)=1-\frac{\omega_{p,b}^{2}}{\omega^{2}-\omega_{0,b}^{2}+i\gamma_{b}\omega }-\frac{\omega_{p}^{2}}{\omega^{2}+i\gamma_{f}\omega }$$

$$\omega_{p}={\left(Ne^{2}/\varepsilon_{0}m^{*}\right)}^{1/2}$$

In the model, $\omega_{p}$ is the plasma frequency, e is the electron charge (1.602 × 10^−19^ C), *ε_0_* presents the permittivity of free space (8.85 × 10^−12^ F/m), $m^{*}$ is the effective electron mass (0.35 × 9.1 × 10^−31^ kg), N is the free electron density (3.6 × 10^20^ cm^−3^), $\gamma_{b}$ is the bound electron damping, $\gamma_{f}$ is the effective free electron damping, $\omega_{p,b}$ is the bound electron plasma frequency, $\omega_{0,b}$ is the resonance frequency. According to the Drude model, the dielectric constant is mainly determined by $\omega_{p}$ and N, and the changes in both are caused by the formation of structural defects and the interaction between ITO and TiN interfaces.

To further investigate the nonlinear absorption properties of TiN/ITO composite, this study mainly focuses on investigation of the optical nonlinearity of TiN/ITO composite films in the near-infrared region. As we all know, ENZ materials [26,27]—|Re{ε}| < 1 materials or spectral regions—are the newest additions to the catalogue of nonlinear materials with special properties such as large refractive index tuning [17,28], enhanced harmonic generation [29], and approximate generation of phase-conjugate waves [30] in their ENZ regions. According to Figure 4, the real part of the dielectric constant of TiN thin films cannot meet the ENZ condition at 1550 nm. As is well known, the optical nonlinearity of ITO films is mainly caused by their ENZ region [17]. Therefore, in the experiment, by introducing an ITO thin film layer, the TiN/ITO composite film can meet the ENZ condition at around the laser wavelength of 1550 nm. According to Figure 4, with the thickness of the ITO film increasing, the ENZ real part of the TiN/ITO composite film varies from 1895 nm to 935 nm. It is well known that the dispersion relation of TCO is related to the carrier concentration of deposited films [31]. As the thickness of the ITO film increases, the ITO film has better crystallinity and the square resistance decreases with the increase in carrier concentration inside the film, and the change in carrier concentration leads to an increase in the plasma resonance frequency. The synergistic effect between ITO and TiN films is also responsible for the variation of ENZ wavelengths in composite films [32]. In addition, the introduction of defects in TiN films is also the part reason for the change in the dielectric constant of composite films. By combining TiN/ITO and changing the plasma frequency, the ENZ wavelength tuning of TiN thin films can be achieved, which will also enhance the nonlinear optical performance of TiN/ITO composite films. The plasmon resonance frequency became larger with the thickness of ITO layer increasing. That is, the real part of ENZ wavelength showed a blue-shift.

To further investigate the nonlinear absorption properties of TiN/ITO composite samples, the Z-scan technique was employed for characterization. The Z-scan theory is used for obtaining nonlinear absorption coefficient and theoretical fits [32,33,34]. Under nonlinear conditions, the total nonlinear absorption coefficient of the sample, denoted as α, can be expressed as α(*I*) = α_o_ + β(*I*)*I*, where *I*, *α_o_*, and *β*(*I*) represent the incident light intensity, linear absorption coefficient, and nonlinear absorption coefficient, respectively. Consequently, the propagation equation for the sample can be formulated as:

$$\alpha \left(I\right)=\alpha_{0}\frac{1}{1+I_{0}/I_{s}}+\beta I$$

When the aperture A is in an open state, the normalized transmittance at a distance z from the focus is:

$$T_{\mathrm{norm}}=1-\frac{1}{2\sqrt{2}}\frac{\beta I_{o}L_{eff}}{1+(\frac{z}{z_{o}}{)}^{2}}$$

Here, *z* represents the linear distance between the sample and the focus, $z_{0}=\frac{k\omega_{0}^{2}}{2}$ is the Rayleigh length, k is the wavenumber, and $\omega_{0}$ is the beam radius at the 1e2 level of the intensity distribution. For the diffraction length of the Gaussian beam, *I*_o_ denotes the laser intensity at the focus. The expression Leff=1−exp−α0Lα0 represents the effective optical length of the sample, where L is the thickness of the thin film sample.

**Figure 5:**
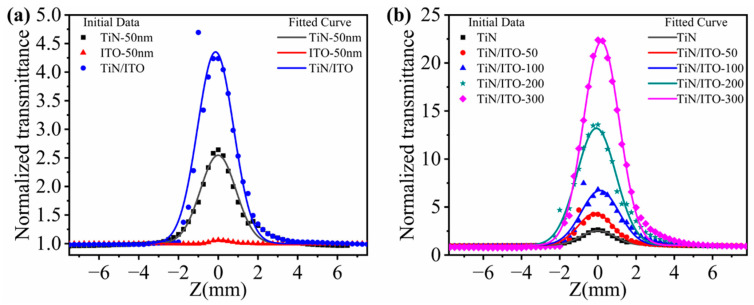
The normalized transmittance of (**a**) single-layer TiN, single-layer ITO, and TiN/ITO composite films. (**b**) single-layer TiN and TiN/ITO composite films with different ITO thicknesses.

It is well known that near 1550 nm wavelength is a low-loss window for fiber-optic communication, and for this reason we have investigated the nonlinear absorption properties of the samples at a 1550 nm excitation wavelength using the Z-scanning technique. Figure 5 shows the normalized transmittance of all samples measured under the OA Z-scan system with an excitation wavelength of 1550 nm. According to Figure 5, within the OA Z-scan system, under the excitation of the laser, all samples exhibit saturated absorption, with single-layer TiN having a lower absorption intensity. The absorption intensity of single-layer ITO is higher than that of TiN, while the saturated absorption intensity of composite samples is significantly enhanced. We observe an increase in the transmittance of the samples with the thickness of ITO increasing, and the transmittance reaches its peak at the focus. Among them, the highest normalized transmittance reaches 22.40. Furthermore, the transmittance of TiN films with an ITO thin film as a buffer layer is higher than that of films without ITO. This indicates that ITO can enhance the nonlinear optical properties of titanium nitride. From the curve in Figure 5, we observe that the normalized transmittance of all ITO thin films also increases with increasing thickness, reaching a maximum of 3.55. Notably, the transmittance is lower than that of TiN film samples with the same thickness of ITO as the substrate. Through a comparative analysis of Figure 5, it becomes evident that the saturation absorption (SA) of the TiN/ITO composite films is stronger than that of single-layer samples. The primary reason for this is the local electric field enhancement arising from the strong coupling between ITO and TiN.

Based on the Z-scan theory, the nonlinear absorption coefficient β values of all thin-film samples were obtained through fitting, as shown in Figure 5. From the figure, it is easy to analyze and observe that the nonlinear absorption coefficient of the TiN/ITO films increases with the thickness of the ITO film, ranging from −2.15 × 10^−5^ cm/W to −2.97 × 10^−4^ cm/W. Compared to the single-layer titanium nitride film, this represents a 14.02-fold. Meanwhile, the absolute value of the nonlinear absorption coefficient of the ITO films initially decreases, from −6.42 × 10^−7^ cm/W to −6.19 × 10^−7^ cm/W, and then increases after the ITO thickness exceeds 100 nm. The nonlinear absorption coefficient reaches its maximum value of −3.13 × 10^−5^ cm/W when the ITO thickness is 300 nm.

**Figure 6:**
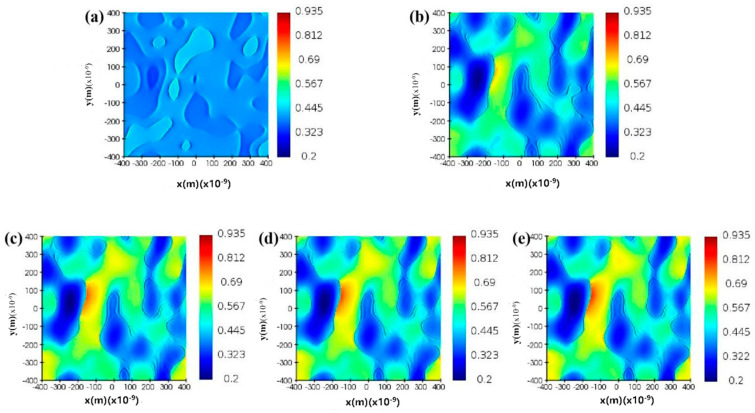
FDTD simulation patterns of (**a**) single-layer TiN, (**b**) TiN/ITO composite films with an ITO thickness of 50 nm, (**c**) TiN/ITO composite films with an ITO thickness of 100 nm, (**d**) TiN/ITO composite films with an ITO thickness of 200 nm, (**e**) TiN/ITO composite films with an ITO thickness of 300 nm.

In order to verify the optical nonlinearity of the sample, FDTD simulation was used to simulate the electric field distribution of TiN/ITO structure at a nonlinear Z-scan excitation wavelength of 1550 nm. On the x-y plane, we selected a 400 nm × 400 nm rectangle as the simulation area. The bottom layer is a glass substrate, the middle layer is ITO films with different thicknesses, and the top layer is TiN with a thickness of 50 nm. In the calculation, we used a 1550 nm wavelength laser perpendicular to the x-y plane of TiN and TiN/ITO systems. The polarization along the y-axis, the dielectric constant, roughness, and other parameters used in the simulation are all consistent with those of experimental test data. Accord to the figure, it can be seen that the surface of the single-layer TiN film is dense and uniform, with an electric field strength of about 0.682. However, TiN/ITO composite films exhibit greater roughness, with local electric field strength increased to 0.935. For ease of comparison, the electric field intensity distribution at 1550 nm wavelength is selected within the same range. From Figure 6, we can see that compared to the single-layer TiN film, the electric field of TiN/ITO composite film is overall enhanced. However, there is still a strong interaction between ITO and TiN, mainly because the sample still meets the ENZ characteristic condition at the excitation wavelength of 1550 nm. In addition, it can be seen from the graph that the electric field strength of TiN/ITO composite materials increases with the increase of ITO film thickness. This is because the coupling between TiN and ITO increases with the increase of ITO thickness. The trend of electric field variation is consistent with the trend of nonlinear saturation absorption coefficient variation, indirectly proving the influence of ITO thickness on the nonlinear optical properties of composite film samples. 

## 4. Conclusions

In summary, TiN with an ITO buffer layer was fabricated. The tunability of nonlinear optical absorption were induced by the ITO buffer layer. The nonlinear absorption of TiN/ITO composite thin films were enhanced and the ENZ wavelength were shown a blue-shift with the thickness of ITO thin film increasing, which is due to the strong coupling between ITO and TiN. The time-domain finite difference results demonstrated a stronger local electric field in the TiN/ITO composite thin films than in the monolayer TiN film. These results indicate that the synthesized TiN nanostructure-based films have potential applications in nonlinear optical devices.

## Data Availability

Data of the results presented in this article are available from the corresponding author upon reasonable request.
